# Supplementary material for: The DUX-25 after Twenty-Five Years: New Analyses and Reference Data
Source: Children (Basel). 2022 Oct 17;9(10):1569. doi: 10.3390/children9101569 (PMC9600854; doi:10.3390/children9101569)
Supplement: Supplementary file 1 [file children-09-01569-s001.zip › Supplementary File_S1_DUX_25_child English version.pdf]

DUX 25

We want to know how you felt lately.
On every question, can you tell us how that feeling was?
Put a circle around the face that fits best.
With every question you can choose one of the five faces.

This is an example.

**If you felt very happy, put a cicrle around the very happy face** 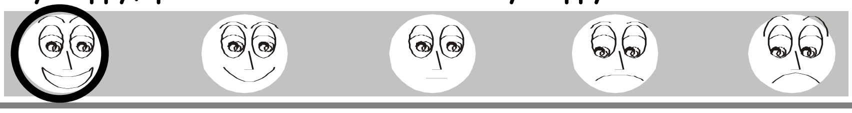
**If you felt very sad, put a circle around the very sad face** 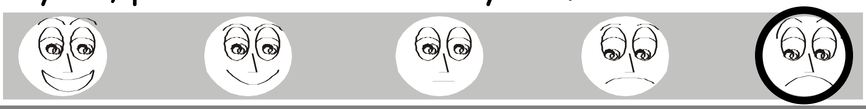
**If you felt normal, put a circle around the normal face** 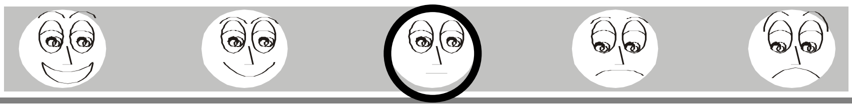
**If you felt a bit happy, put a cicle around the bit happy face** 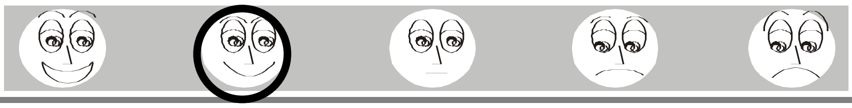
**If you felt a bit sad, put a circle around the bit sad face** 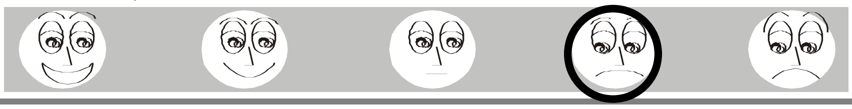

You cannot answer wrongly.
It is important to give your own view.

Succes!

	How do you feel?
1	At school, I feel … 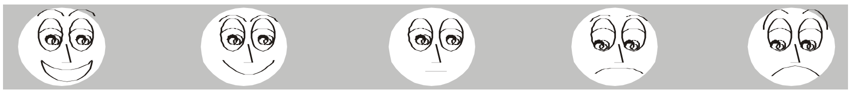
2	I often feel … 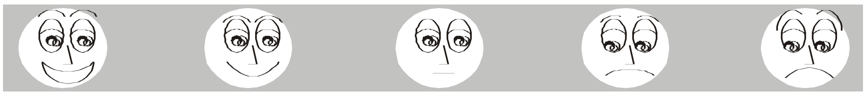
3	Towards grown-ups, I am usually ... 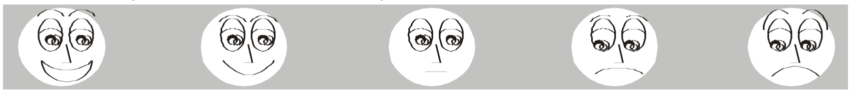
4	Right now, I feel … 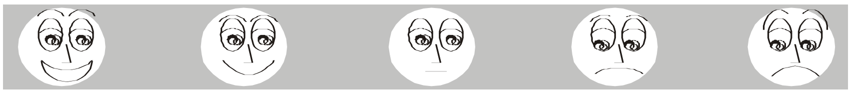
5	With regards to the children in my class, I feel … 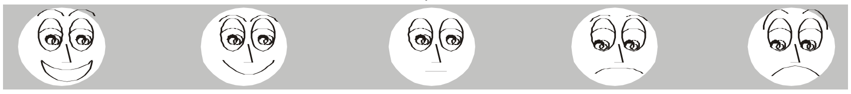
6	At home, I am usually … 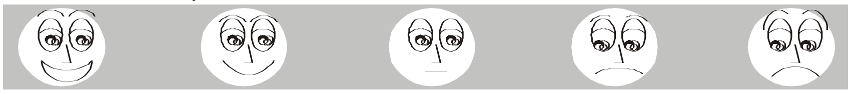
7	With regards to my Dad, I feel … 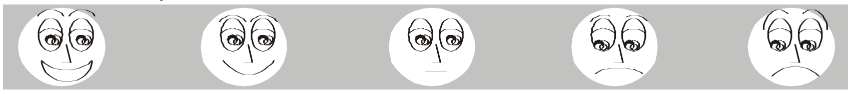
8	About other people, I tend to feel … 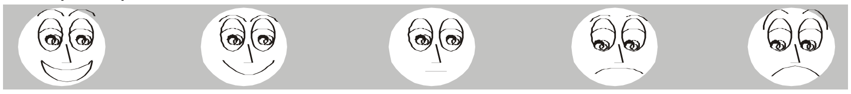
9	About my stamina, I feel … 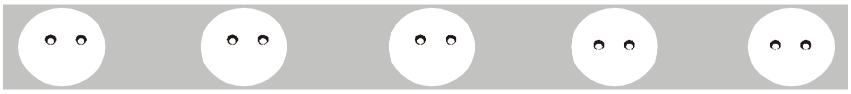
10	About the things I do, I am … 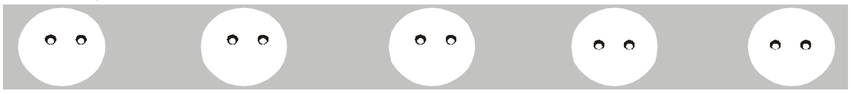

How do you feel?	
11	About the way I look, I am … 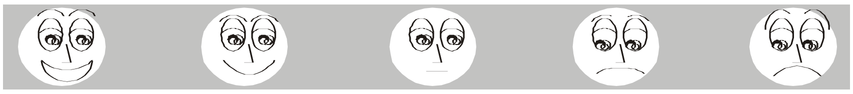
12	When I look at other children, I often feel … 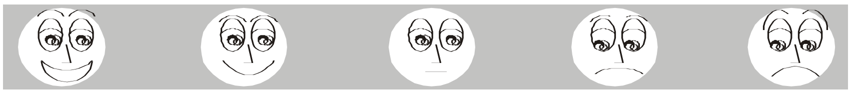
13	About my body, I feel … 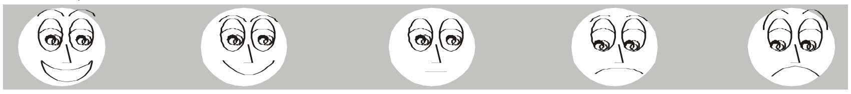
14	When I wake up, I tend to be … 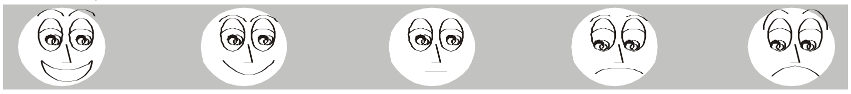
15	About my life, I feel … 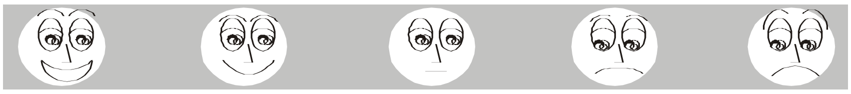
16	About my friends I feel … 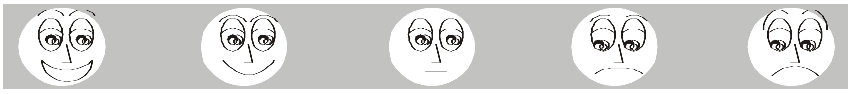
17	The things I think about are often … 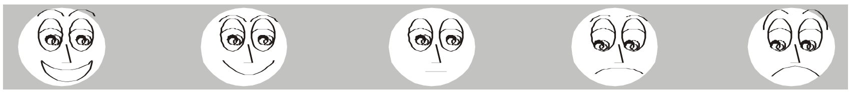
18	About the things we do together at home, I am … 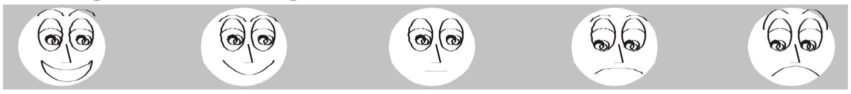
19	With regards to my Mum, I feel … 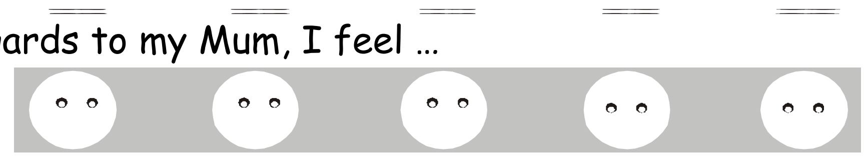
20	About the teachers at school, I feel … 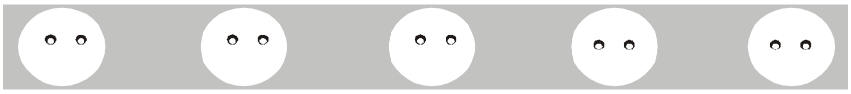

	How do you feel?
21	About how tall I am, I feel … 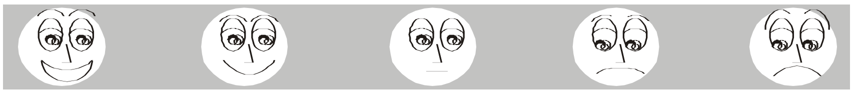
22	About my schoolwork, I feel … 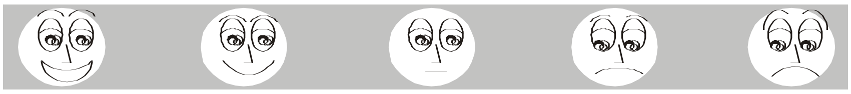
23	About my weight, I feel … 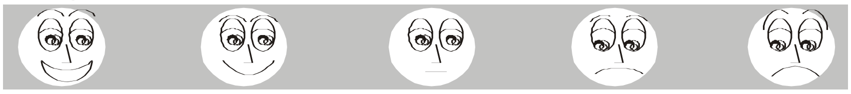
24	To do things together with somebody else is … 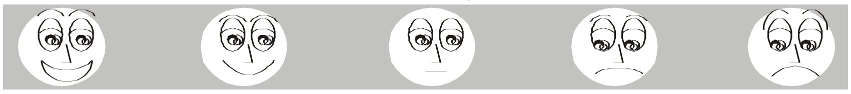
25	In bed at night I usually feel… 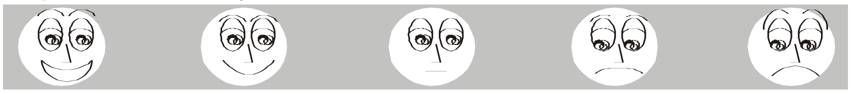

Three more questions:

26	How are you? 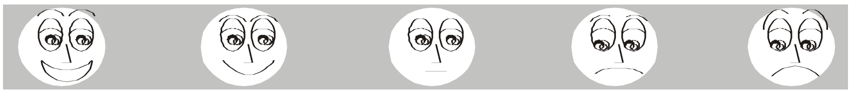
27	What do you think of your health? 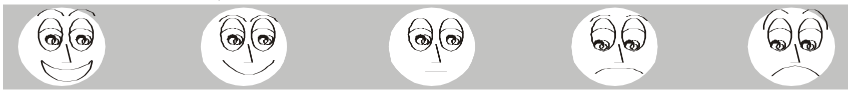
28	What do you think of these questions? 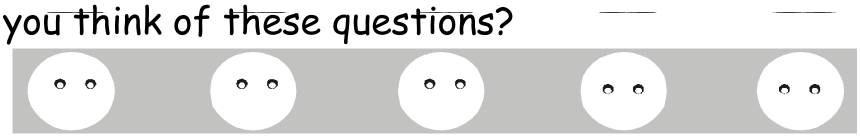

Thanks a lot!
