# Supplementary material for: The DUX-25 after Twenty-Five Years: New Analyses and Reference Data
Source: Children (Basel). 2022 Oct 17;9(10):1569. doi: 10.3390/children9101569 (PMC9600854; doi:10.3390/children9101569)
Supplement: Supplementary file 1 [file children-09-01569-s001.zip › Supplementary File_S3_DUX25_Manova_Subscales_gender_age.pdf]

Supplemental Table S3. MANOVA and corresponding ANOVA's, DUX-25 Subscales by Gender and Age Group

Multivariate effects	F(5, 585)	Wilks/
**Gender**	4.47***	.96
**Age Group**	8.05***	.94
**Gender x Age Group**	1.99	.98

							**Univariate effects**					
	**Boys**			**Girls**			**Gender**		**Age Group**		**Gender x Age Group**	
**DUX-25 Subscale**	***M***	***SD***	***N***	***M***	***SD***	***N***	F(1, 589)	$÷^{2}_{partial}$	F(1, 589)	$÷^{2}_{partial}$	F(1, 589)	$÷^{2}_{partial}$
**Emotion**												
Total	76.70	14.79	256	75.70	16.24	337	0.62	.00	7.71**	.01	0.64	.00
8 to 12 years	78.38	14.11	92	78.40	14.15	145						
13 to 17 years	75.76	15.11	164	73.66	17.42	192						
**Social Close**												
Total	86.39	13.78	256	86.99	13.42	337	0.35	.00	2.76	.00	0.81	.00
8 to 12 years	86.96	14.05	92	88.68	12.56	145						
13 to 17 years	86.08	13.66	164	85.72	13.92	192						
**Social Far**												
Total	73.78	15.75	256	74.57	13.80	337	0.16	.00	10.94**	.02	0.00	.00
8 to 12 years	76.43	14.94	92	76.90	13.79	145						
13 to 17 years	72.29	16.05	164	72.82	13.58	192						
**Home**												
Total	87.48	13.76	256	88.01	14.32	337	0.02	.00	22.65***	.04	0.04	.00
8 to 12 years	90.92	11.46	92	91.34	11.29	145						
13 to 17 years	85.55	14.58	164	85.49	15.81	192						
**Body**												
Total	82.10	15.80	256	76.45	18.91	337	13.82***	.02	30.12***	.05	7.04**	.01
8 to 12 years	84.74	15.46	92	83.19	14.59	145						
13 to 17 years	80.61	15.84	164	71.35	20.20	192						

Note: * p < .05, ** p < .01, *** p < .001
