# Supplementary material for: The DUX-25 after Twenty-Five Years: New Analyses and Reference Data
Source: Children (Basel). 2022 Oct 17;9(10):1569. doi: 10.3390/children9101569 (PMC9600854; doi:10.3390/children9101569)
Supplement: Supplementary file 1 [file children-09-01569-s001.zip › Supplementary File_S4_DUX25_MultiGroup_CFA_Gender.pdf]

Supplemental Table S4. Multigroup Comparison, 5 Confirmatory Factor Model for Gender

Model	Df	Chi-square	RMSEA	SRMR	CFI	Change CFI	Different?
Both Gender Groups (n = 593)	265	914.58	.064	.059	.878	n/a	n/a
Girls (n = 337)	265	723.40	.072	.064	.858	n/a	n/a
Boys (n = 256)	265	554.79	.065	.065	.871	n/a	n/a
Configural Invariance	530	1278.23	.069	.064	.863	n/a	n/a
Metric Invariance	550	1303.42	.068	.068	.862	.001	No
Scalar Invariance	570	1363.40	.069	.070	.855	.007	No
Strict Invariance	595	1417.54	.068	.072	.850	.005	No

Note: Models for Boys and Girls are non-significantly different up till and including the highest level of comparison (Strict Invariance)
