# Supplementary material for: The DUX-25 after Twenty-Five Years: New Analyses and Reference Data
Source: Children (Basel). 2022 Oct 17;9(10):1569. doi: 10.3390/children9101569 (PMC9600854; doi:10.3390/children9101569)
Supplement: Supplementary file 1 [file children-09-01569-s001.zip › Supplementary File_S5_DUX_25_MultiGroup_CFA_Age_Groups_Girls.pdf]

Supplementary Table S5. Multigroup Comparison 5 Confirmatory Factor Model for Age Groups for Girls

Model	Df	Chi-square	RMSEA	SRMR	CFI	Change CFI	Different?
Both Age Groups (n = 337)	265	723.40	.072	.064	.858	n/a	n/a
8 to 12 years (n = 145)	265	475.17	.074	.080	.802	n/a	n/a
13 to 17 years (n = 192)	265	557.36	.076	.069	.860	n/a	n/a
Configural Invariance	530	1032.54	.075	.074	.841	n/a	n/a
Metric Invariance	550	1062.32	.074	.081	.838	.003	No
Scalar Invariance	570	1122.33	.076	.083	.825	.013	Yes
partial Scalar Invariance[a]	569	1088.48	.074	.082	.835	.003	No
Strict Invariance	594	1235.24	.080	.088	.797	.038	Yes
Partial Strict Invariance[b]	587	1125.24	.074	.084	.829	.006	No

[a] Intercept (mean) for item 20 (teachers) is set free to vary across age groups.

[b] Variances for items 2, 5, 7, 9, 13, 17 and 19 are set free to vary across age groups.
