# Supplementary material for: The DUX-25 after Twenty-Five Years: New Analyses and Reference Data
Source: Children (Basel). 2022 Oct 17;9(10):1569. doi: 10.3390/children9101569 (PMC9600854; doi:10.3390/children9101569)
Supplement: Supplementary file 1 [file children-09-01569-s001.zip › Supplementary File_S6_DUX_25_MultiGroup_CFA_Age_Groups_Boys.pdf]

Supplementary Table S6. Multigroup Comparison, 5 Confirmatory Factor Model for Age Groups for Boys

**Model**	**Df**	**Chi-square**	**RMSEA**	**SRMR**	**CFI**	**Change CFI**	**Different?**
Both Age Groups (n = 256)	265	554.79	.065	.065	.871	n/a	n/a
8 to 12 years (n = 92)	265	468.89	.091	.090	.761	n/a	n/a
13 to 17 years (n = 164)	265	473.82	.069	.074	.863	n/a	n/a
Configural Invariance	530	942.71	.078	.080	.827	n/a	n/a
Metric Invariance	550	973.79	.078	.083	.822	.005	No
Scalar Invariance	570	1021.09	.079	.085	.811	.011	Yes
Partial Scalar Invariance[a]	569	1021.49	.078	.085	.814	.008	No
Strict Invariance	594	1095.55	.081	.092	.789	.025	Yes
Partial Strict Invariance[b]	591	1054.75	078	.083	.805	.009	No

[a] Intercept (mean) for item 17 (things I think) is set free to vary across age groups.

[b] Variances for items 15, 19 and 21 are set free to vary across age groups
