# Supplementary material for: The DUX-25 after Twenty-Five Years: New Analyses and Reference Data
Source: Children (Basel). 2022 Oct 17;9(10):1569. doi: 10.3390/children9101569 (PMC9600854; doi:10.3390/children9101569)
Supplement: Supplementary file 1 [file children-09-01569-s001.zip › Supplementary File_S7_DUX_25_MultiGroup_CFA_Children_Parents.pdf]

Supplementary Table S7. Multigroup Comparison, 5 Factor Model for Children and Parents

Model	Df	Chi-square	RMSEA	SRMR	CFI	Change CFI	Different?
Both Children and Parents (n = 1361)	265	1980.90	.069	.057	.882	n/a	n/a
Children 8 to 17 year (n = 593)	265	914.58	.064	.059	.878	n/a	n/a
Parents (n = 768)	265	1507.55	.078	.063	.871	n/a	n/a
Configural Invariance	530	2422.17	.072	.061	.874	n/a	n/a
Metric Invariance	550	2472.04	.072	.064	.872	.002	No
Scalar Invariance	570	2697.37	.074	.067	.858	.014	Yes
Partial Scalar Invariance[a]	568	2605.34	.073	.066	.864	.008	No
Strict Invariance	593	2801.58	074	.068	.852	.012	Yes
Partial Strict Invariance[b]	592	2756.76	073	.068	.855	.009	No

[a] Intercepts (means) for item '6, at home' and '8, other people' are set free to vary across groups

[b] Variance for item '9, stamina' is set free to vary across child and parent group
